# Successful Hemispherotomy in a Patient With 22q11.2 Deletion Syndrome Who Had Developmental and Epileptic Encephalopathy With Spike-and-Wave Activation During Sleep

**DOI:** 10.7759/cureus.58424

**Published:** 2024-04-16

**Authors:** Naoki Yamada, Ichiro Kuki, Takehiro Uda, Shin Okazaki

**Affiliations:** 1 Pediatric Neurology, Osaka City General Hospital, Osaka, JPN; 2 Neurosurgery, Osaka Metropolitan University, Osaka, JPN

**Keywords:** pediatric, hemispherotomy, polymicrogyria, drug-resistant epileps, 22q11.2 deletion sydrome

## Abstract

We report a case of developmental and epileptic encephalopathy with spike-and-wave activation during sleep with 22q11.2 deletion syndrome in a patient who had undergone hemispherotomy and achieved developmental improvement. A four-year-old male child with paralysis on the left side of his body since birth had a mild developmental delay. An MRI of the brain revealed polymicrogyria diffusely throughout the right hemisphere. He was diagnosed with the 22q11.2 deletion syndrome at one year of age. Focal impaired awareness seizure in the right hemisphere origin and focal to bilateral tonic-clonic seizure appeared by two years of age. At three years of age, myoclonic seizures occurred, which induced frequent falls. Simultaneously, developmental and epileptic encephalopathy with spike-and-wave activation during sleep were observed. At four years and seven months of age, the patient underwent a right hemispherotomy. Epileptic seizures and spike-and-wave activation during sleep disappeared, and cognitive improvement was observed one year after surgery. In spite of chromosomal abnormalities being present, drug-resistant epilepsy with localized regions on MRI should be evaluated to determine surgical options to improve cognitive function and development.

## Introduction

The 22q11.2 deletion syndrome (22q11.2 DS) is the most common chromosomal microdeletion syndrome [1]. The risk of acute symptomatic seizures is high in these patients. Hypocalcemia due to hypoparathyroidism and exposure to psychotropic drugs are the main causes of acute symptomatic seizure [2]. The prevalence of febrile seizures is also higher in patients with 22q11.2 DS than in the general population [3]. Although few studies have focused on epilepsy in these patients, the prevalence of epilepsy has been reported to be approximately 10% [3].

Developmental and epileptic encephalopathy with spike-and-wave activation during sleep (DEE-SWAS) is a childhood epilepsy syndrome. The longer the duration of spike-and-wave activation in sleep (SWAS), the worse the neuropsychological sequelae [4]. Prevention of poor long-term cognitive outcomes is achieved through seizure control and termination of SWAS. Here, we report a case of DEE-SWAS with 22q11.2 DS in which seizures disappeared and development improved after hemisphere dissection.

## Case presentation

**Figure 1:**
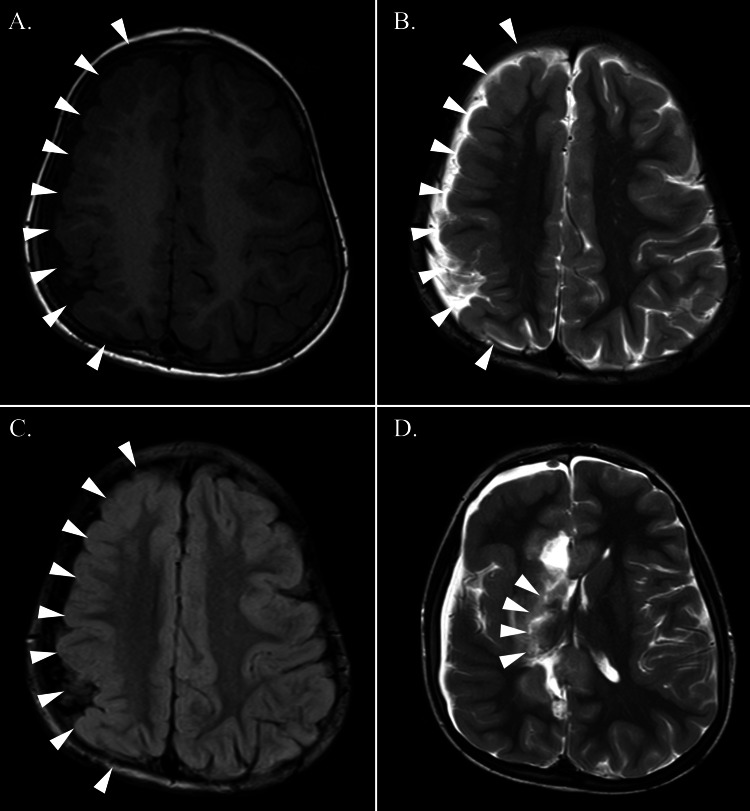
Pre-surgical and post-surgical MRI findings. Pre-surgical T1-weighted MRI (A), T2-weighted MRI (B), and FLAIR imaging (C) at four years of age. MRI shows diffuse right hemisphere polymicrogyria and atrophy (arrowhead), and normal findings in the left hemisphere. (D) Post-surgical T2-weighted MRI shows a disconnection line (arrowhead). FLAIR: fluid-attenuated inversion recovery

A four-year-old boy had been paralyzed on the left side of his body since birth and had mild developmental delay. Although he could walk alone, he could not climb stairs alone. he had no abnormalities in his perinatal history. The patient had no history of febrile seizures or hypocalcemia. He had no history of congenital heart disease. He had no craniofacial abnormalities. An MRI of the brain revealed polymicrogyria in the diffuse left hemisphere, and the right hemisphere was normal (Figures 1A-1C).

**Figure 2:**
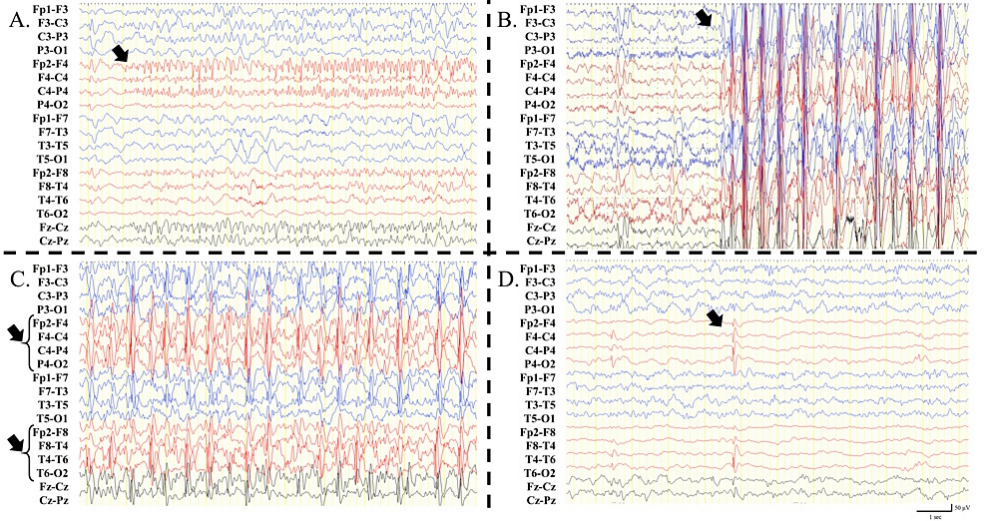
Ictal EEG findings of focal impaired awareness seizure (A) and myoclonic seizures (B). EEG findings of non-rapid eye movement sleep at three years of age (C), and one year postoperatively. (A) Epileptic discharge arises from the right hemisphere (arrow); (B) myoclonic seizures consistent with diffuse poly spike and wave activities (arrow); (C) continuous diffuse spike and wave activities with higher amplitude over the right hemisphere (arrows); (D) disappearance of the spike and wave activities and existence of a right hemisphere isolated spike (arrow). EEG: electroencephalogram

At one year of age, 22q11.2 deletion was confirmed by fluorescence in situ hybridization. Focal to bilateral tonic-clonic seizure (FBTCS) occurred when the patient was one year and three months old. His seizure started with brief episodes of psychomotor arrest followed by a left deviation of the eyes. Although levetiracetam was initiated, FBTCS recurred monthly. A 24-hour video electroencephalogram (EEG) captured focal impaired awareness seizures originating in the right hemisphere 20 times per day, and focal interictal epileptiform discharges were frequently observed at F4 and C4 (Figure 2A).

At three years of age, myoclonic seizures occurred, and their frequency increased up to several times per hour (Figure 2B). Myoclonic seizures induced frequent falls and traumatic injuries sometimes occurred. Simultaneously, continuous diffuse spike-and-wave activities during non-rapid eye movement sleep were observed (Figure 2C). The epileptic discharge voltage was consistently high on the right side and began slightly earlier on the right side. With worsening EEG findings, the patient could not speak sentences and used only words to express thoughts. Simultaneously, his reactivity became scarce, and cognitive decline appeared. Several anti-seizure medicines (valproic acid, lacosamide, clobazam, topiramate, perampanel, ethosuximide, and clonazepam) could not control the myoclonic seizures, and SWAS persisted.

At four years and seven months of age, the patient underwent right hemispherotomy (Figure 1D). We chose the vertical approach, and no surgical complications occurred. The myoclonic seizures and FBTCS disappeared (Engel class I) one year postoperatively. Postoperative EEG data showed the disappearance of SWAS and focal spikes on the disconnected side (Figure 2D). Anti-seizure medicines could be reduced from three drugs (valproic acid, clobazam, and ethosuximide) at the preoperative stage to valploic acid only after one year postoperatively. Cognition improved postoperatively, and the patient could speak in full sentences again.

## Discussion

In 22q11.2 DS, DEE-SWAS is extremely rare, and only one patient was reported to have bilateral polymicrogyria who then underwent anterior callosotomy [5]. Focal resection, including hemispherotomy, in 22q11.2 DS has not been previously reported. In half of the patients with 22q11.2 DS who had epilepsy, neurological findings reported included polymicrogyria, hippocampal malrotation, heterotopia, and diffuse cerebral atrophy, while other patients showed no obvious abnormalities [6]. In the present case, although the etiology due to chromosomal abnormalities could not be ruled out, we performed a right hemispherotomy, considering the MRI and EEG findings. The surgery resulted in an Engel class I outcome and improved cognition.

DEE-SWAS causes deterioration of cognitive function and developmental delays, and severe neuropsychological sequelae are common [4]. Treating seizures using medicines such as valproic acid, ethosuximide, and corticosteroids is well-established; however, drug-resistant cases are frequent. Although the effect of surgical treatment on idiopathic DEE-SWAS is controversial, the effectiveness of callosotomy and resective surgery in drug-resistant symptomatic DEE-SWAS has been previously reported [5].

Patients with 22q11.2 DS are at an increased risk of neurodevelopmental disorders [7], which is further elevated in patients with epilepsy [3]. Epilepsy-induced aberrant synaptic plasticity and a subsequent imbalance in neuronal excitation/inhibition are considered to be involved in neurodevelopmental problems [8]. Early intervention in epileptic activity is crucial because the vulnerable central nervous systems of individuals with 22q11.2 DS may deteriorate due to epilepsy.

## Conclusions

Patients with 22q11.2 DS have a high probability of developing epilepsy with or without brain malformation. Here, we demonstrate a successful hemispherotomy in a patient with 22q11.2 DS who had polymicrogyria in the right hemisphere and drug-resistant epilepsy. Despite underlying chromosomal abnormalities, drug-resistant epilepsy with localized regions on MRI should be considered to evaluate the surgical options for early improvement in cognitive function and development.
